# Combining information from a clinical data warehouse and a pharmaceutical database to generate a framework to detect comorbidities in electronic health records

**DOI:** 10.1186/s12911-018-0586-x

**Published:** 2018-01-24

**Authors:** Emmanuelle Sylvestre, Guillaume Bouzillé, Emmanuel Chazard, Cécil His-Mahier, Christine Riou, Marc Cuggia

**Affiliations:** 1 INSERM, U1099, F-35000 Rennes, France; 2 Université de Rennes 1, LTSI, F-35000 Rennes, France; 3 CHU Rennes, CIC Inserm 1414, F-35000 Rennes, France; 4 CHU Rennes, Centre de Données Cliniques, F-35000 Rennes, France; 5 Département de Santé Publique, Université de Lille EA 2694, CHU Lille, F-59000 Lille, France

**Keywords:** Databases, Pharmaceutical, Clinical data warehouse, Comorbidity, Billing codes, Drug prescriptions, Laboratory test results

## Abstract

**Background:**

Medical coding is used for a variety of activities, from observational studies to hospital billing. However, comorbidities tend to be under-reported by medical coders. The aim of this study was to develop an algorithm to detect comorbidities in electronic health records (EHR) by using a clinical data warehouse (CDW) and a knowledge database.

**Methods:**

We enriched the Theriaque pharmaceutical database with the French national Comorbidities List to identify drugs associated with at least one major comorbid condition and diagnoses associated with a drug indication. Then, we compared the drug indications in the Theriaque database with the ICD-10 billing codes in EHR to detect potentially missing comorbidities based on drug prescriptions. Finally, we improved comorbidity detection by matching drug prescriptions and laboratory test results. We tested the obtained algorithm by using two retrospective datasets extracted from the Rennes University Hospital (RUH) CDW. The first dataset included all adult patients hospitalized in the ear, nose, throat (ENT) surgical ward between October and December 2014 (ENT dataset). The second included all adult patients hospitalized at RUH between January and February 2015 (general dataset). We reviewed medical records to find written evidence of the suggested comorbidities in current or past stays.

**Results:**

Among the 22,132 Common Units of Dispensation (CUD) codes present in the Theriaque database, 19,970 drugs (90.2%) were associated with one or several ICD-10 diagnoses, based on their indication, and 11,162 (50.4%) with at least one of the 4878 comorbidities from the comorbidity list. Among the 122 patients of the ENT dataset, 75.4% had at least one drug prescription without corresponding ICD-10 code. The comorbidity diagnoses suggested by the algorithm were confirmed in 44.6% of the cases. Among the 4312 patients of the general dataset, 68.4% had at least one drug prescription without corresponding ICD-10 code. The comorbidity diagnoses suggested by the algorithm were confirmed in 20.3% of reviewed cases.

**Conclusions:**

This simple algorithm based on combining accessible and immediately reusable data from knowledge databases, drug prescriptions and laboratory test results can detect comorbidities.

**Electronic supplementary material:**

The online version of this article (10.1186/s12911-018-0586-x) contains supplementary material, which is available to authorized users.

## Background

Medical coding [1] is used for a variety of activities, from observational studies to hospital billing [2, 3]. Therefore, medical codes should match as much as possible the information of the patient’s medical record [4]. However, clinical coders do not fill in medical records or take care of patients. Several studies demonstrated that the agreement between medical records and medical codes is variable, if not suboptimal [5], and that comorbidities, particularly, tend to be under-reported. In this context, the term comorbidities includes all diagnoses beside the principal diagnosis (i.e., the main reason for the patient’s hospitalization) that required specific diagnostic and/or therapeutic interventions during the hospital stay [6]. Yet, codes are used on the basis of the assumption that they contain detailed clinical and outcome data [7, 8]. For this, clinical coders need to have access to all the data contained in the medical record.

With the increasing use of electronic health records (EHR), such data become more easily available, especially through clinical data warehouses (CDW) that are great sources of integrated heterogeneous and exhaustive information [9, 10]. In parallel, knowledge databases, such as pharmaceutical databases, are used to analyze and process clinical data and can also support clinical decision-making [11, 12]. The challenge lies in combining these different sources of information to improve the accuracy and exhaustivity of medical codes.

The aim of this study was to develop an algorithm to detect comorbidities in EHR using a clinical data warehouse and knowledge databases. We focused on two different types of data, drug prescriptions and laboratory tests, to assess how they contribute and complement each other for identifying comorbidities.

## Methods

### Data sources

We used three data sources: clinical data from the Rennes University Hospital (RUH) CDW, knowledge data from a pharmaceutical database and the French reference list of comorbidities.

#### The RUH CDW (EHOP)

The EHOP [10, 13] CDW contains most of the EHR, including clinical notes, drug prescriptions, laboratory test records and administrative data. Each patient’s EHR includes all information about his/her hospital stay. If the patient was hospitalized several times, all these hospital stays are grouped in a single EHR. Diagnoses are coded using the French version of the international classification of diseases, 10th edition (ICD-10), that incorporates the specific French modifications. Data are de-identified and subdivided in structured (drug prescriptions, laboratory test records…) and unstructured (clinical notes) data. EHOP does not include records from the Anesthesiology Department and the Intensive Care Unit (available only in paper form) and the nursing files.

#### Theriaque database

The Theriaque database [14] is one of the four drug databases [15] certified by the French Health Authorities (specifically by the Haute Autorité en Santé) and provides exhaustive information on drugs with a marketing authorization. For each drug, this database gives one or more indications (ICD-10 codes, when possible), its Anatomical Therapeutic Chemical (ATC) code [16, 17] and an unique identifier called “Common Unit of Dispensation” (CUD) (“Unité Commune de Dispensation” in French). CUD codes are the French standard for drug identification and are used to process drug data in the Hospital Information System.

#### The french comorbidity list

The French Comorbidity List (“Liste des Comorbidités et Morbidités Associées”) is a reference list from the French Program of Medicalization of the Information Systems (Programme de Médicalisation des Systèmes d’Information) that is based on the Diagnosis-Related-Group (DRG) system. The Program of Medicalization of Information Systems takes comorbidities into account for hospital reimbursement and the major comorbid conditions are identified in this reference list. The aim of this list is to identify comorbidities (ICD-10-coded) with a financial impact on the patient’s hospital stay.

### Algorithm development

**Fig. 1:**
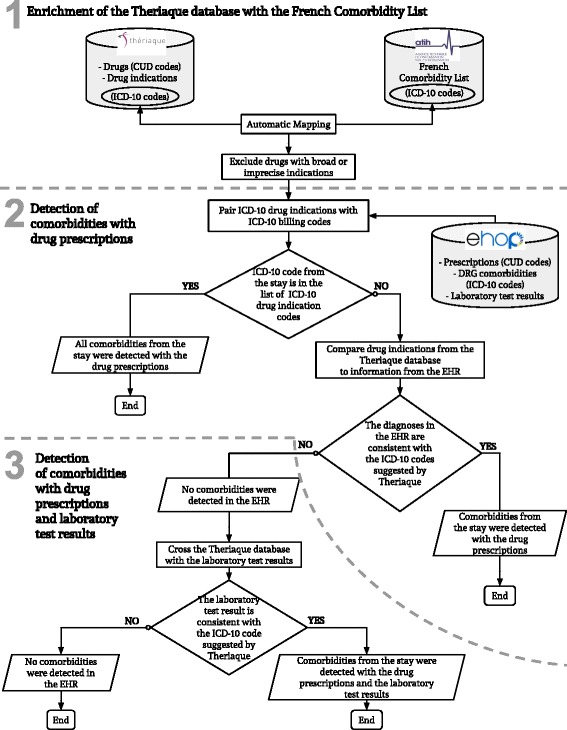
Three-step algorithm. CUD: Common unit of Dispensation (Drug unique identifier). DRG: Diagnosis-Related-Group. EHR: Electronic Health Record. ICD-10: International Classification of Diseases, 10th revision

To detect comorbidities, we developed a three-step algorithm that we then tested in a retrospective observational study (Figure 1).

#### Step one: Enrichment of the theriaque database with the comorbidity list

We paired the Theriaque database with the Comorbidity List to identify drugs associated with at least one major comorbid condition. This step also identified ICD-10 codes associated with a drug indication and eliminated all ICD-10 codes that could not be paired with at least one drug indication. It also excluded drugs with very broad (i.e., more than twenty indications) or only imprecise (e.g., “Pain, unspecified”, R52.9) indications, based on their ATC classification (Additional file 1).

#### Step two: Detection of comorbidities in the EHR by using drug prescriptions

##### 2.Pairing the ICD-10 drug indications with the ICD-10 billing codes

The aim of this step was to estimate the proportion of existing ICD-10 codes from the EHR that can be correctly retrieved by the algorithm. For each hospital stay, we retrieved the names of all the prescribed/administered drugs and all ICD-10 codes originally assigned to that hospital stay by the non-medical coding staff. Then, we compared these ICD-10 codes with those suggested by the Theriaque database for each prescribed/administered drug. This step led to the identification of stays where all ICD-10 codes and drug prescriptions were matched.

##### 3.Comparing the theriaque database drug indications with the EHR information

For the stays where not all drug prescriptions were matched with ICD-10 codes, we derived the potentially missing diagnoses using the prescribed drugs. Briefly, each drug prescription without at least one ICD-10 code that matched the drug indications, according to the Theriaque database, was a clue for a possible missing comorbidity. For these drugs, the Theriaque database suggested a list of ICD-10 codes, among which some were included in the Comorbidity List and were thus flagged as possible comorbidities.

#### Step three: Detection of comorbidities in the EHR using drug prescriptions and laboratory test results

In the last step, we improved comorbidity detection by using another source of structured data. For this study, we focused on sodium polystyrene sulfonate (SPS), because this drug is only prescribed in the case of hyperkalemia (ICD-10 code E87.5). As the French Nephrology Society defines hyperkalemia as a plasma potassium level higher than 5.0 mmol/L [18], we compared drug prescriptions and laboratory test results to detect hyperkalemia in all EHR.

### Study populations

We retrospectively tested the algorithm using two different datasets extracted from the EHOP CDW. The first dataset included all stays of adult patients hospitalized at RUH ear, nose, throat (ENT) surgical ward between October and December 2014 and with at least one drug prescription (ENT dataset). We chose this unit because comorbidities unrelated to the cause of hospitalization are rarely mentioned in the surgical record and thus, it represented a good test for our algorithm.

The second dataset included the stays of all adult patients hospitalized at RUH between January and February 2015 and with at least one drug prescription (general dataset).

### Algorithm evaluation

We reviewed all the diagnoses suggested by the algorithm using the data from the medical records extracted from the EHOP CDW, to find written evidence about such comorbidities in the current or past hospital stays.

We evaluated the algorithm performance by computing Precision, Recall and F-Score metrics for the ENT dataset. For the general dataset, we did not compute Recall because the value obtained for the ENT dataset suggested that the algorithm could efficiently detect comorbidities based on the drug prescriptions.

Precision is the ratio between the number of stays with confirmed diagnoses and all stays with suggested diagnoses. Recall is the ratio between the number of stays with confirmed diagnoses and all stays with missing diagnoses. The F-Score is the harmonic mean of Precision and Recall. The detailed description of the evaluation process can be found in Additional file 2.

### Statistical analysis

We described quantitative variables using means and standard deviation and categorical variables using counts and percentages. We calculated the inter-expert agreement using the Cohen’s Kappa (κ) coefficient. We performed all statistical analyses with R 3.3.1 [19].

The suggested diagnoses were confirmed or not by one medical expert for the ENT dataset and by two medical experts for the general dataset. These investigators had a public health background and were familiar with the EHOP CDW and the Program of Medicalization of Information Systems. They also knew well the coding guidelines because they have been involved in coding for a few years before this study. The two experts reviewed all records from the ENT dataset and only a random sample from the general dataset, to estimate the algorithm metrics and inter-observer agreement. We calculated that the size of the general dataset sample required to have a 90% inter-observer agreement rate, with β = 0.80 and α = 0.05, was 285 stays.

For each drug prescription, the two experts had to confirm at least one of the suggested comorbidities (ICD-10 codes) or to reject all of them. To confirm a diagnosis, they compared the title of the suggested ICD-10 code or its synonyms (based on the list of synonyms provided by ICD-10) with the diseases/symptoms recorded in the EHR. If they rejected all diagnoses suggested by the algorithm based on the drug prescriptions (structured data), they examined the discharge notes (unstructured data) to confirm their decision. This was done to ensure that the rejection was based on the absence of data on a given disease/symptom and not on the CDW inability to extract all available data. Any disagreement between experts required a consensus session with a third expert to reach a final decision.

## Results

### Enrichment of the theriaque database with the comorbidity list and identification of the relevant ICD-10 codes

Among the 22,132 CUD codes presents in the Theriaque database, 19,970 (90.2%) were associated with one or more ICD-10 codes, based on the drug indication(s). Moreover, 11,162 (50.4%) CUD codes were associated with at least one of the 4878 comorbidities from the Comorbidity List. Among the 40,000 codes included in the French ICD-10, only 4407 (11.0%) were present in the Theriaque database as a drug indication.

### Study populations

**Fig. 2:**
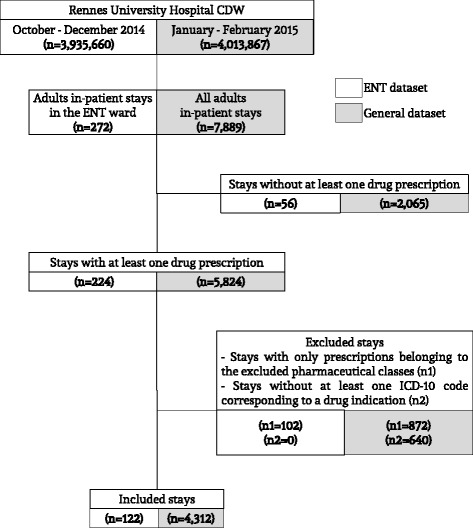
Study flow chart. CDW: Clinical Data Warehouse; ENT: Ear, Nose, Throat; ICD-10: International Classification of Diseases, 10th revision

After excluding all hospital stays without at least one drug prescription, or with prescriptions of excluded drugs or without at least one ICD-10 code associated with a drug, the ENT dataset included 122 stays and the general dataset 4312 stays (Figure 2).

### Inter-observer agreement

We assessed the inter-expert agreement using a random sample (*n* = 285) from the general dataset (see Materials and Methods). The two medical experts reached an agreement on 280 cases (*κ* = 0.74; 95% CI, 0.58–0.90). For the five remaining stays, the experts’ disagreement could be explained by the Program of Medicalization of the Information Systems coding rules. For instance, the reimbursement guidelines do not allow coding some diagnoses/symptoms. Accordingly, even if present in the medical record (and, thus, confirming the suggested comorbid conditions), these diagnoses will not be coded in the hospital stay (and, as a consequence, the drug will not be associated with an ICD-10).

### Detection of comorbidities in the EHR

#### Evaluation of the ENT dataset

Among the 730 ICD-10 billing codes present in the 122 stays of the ENT dataset, 339 (46.4%) were detected by the algorithm. The algorithm suggested a possible missing comorbidity in 92 of the 122 stays (75.4%) (Additional file 3).

The expert confirmed the suggested diagnoses in 41 of these 92 stays (44.6%) and added 58 ICD-10 codes to the stay (Additional file 4). Among these 41 stays, 12 (29.2%) had a major comorbidity (i.e., included in the French Comorbidity List) (Table 1) (Additional file 5). Recall was 95.3% and Precision was 44.6%, with an F-Score of 60.7%.

**Table 1:** Number of stays in which comorbidities (ICD-10 codes) were detected by the algorithm using the drug prescriptions

	ENT dataset		General dataset	
	Stays (*n* = 122)	ICD-10 codes (*n* = 730)	Stays (*n* = 4312)	ICD-10 codes (*n* = 4932)
Comorbidities detected using the drug prescriptions; number (%)	30 (24.5)	339 (46.4)	1362 (31.6)	1271 (25.8)
Missing comorbidities; number (%)	92 (75.4)	–	2950 (68.4)	–
Suggested comorbidities	*n* = 92		n = 285^b^	
Suggested comorbidities, confirmed; number (%)	41 (44.6)	58 (+7.9 ^a^)	58 (20.3)	76 (+6.0 ^a^)
Added major comorbidity; number (%)	12/41 (29.2)	12 (+1.6^a^)	18/58 (31.0)	29 (+2.3 ^a^)

^a^Improvement after code addition. ^b^Random sample from the general dataset. ENT: Ear, Nose, Throat; ICD-10: International Classification of Diseases, 10th revision

The medical expert did not confirm the suggested diagnoses in 51 of the 92 stays for two main reasons: i) the stays did not contain information concerning the suggested comorbidity (*n* = 31; 60.8%); ii) the drug indication was based on implicit judgments and, therefore, could not be added to the stay (*n* = 20; 39.2%).

Drug prescriptions could not be combined with the laboratory test results (step 3) because only one stay in this dataset included a SPS prescription.

#### Evaluation of the general dataset

Among the 4932 ICD-10 billing codes present in the 4312 stays of the general dataset, 1271 (25.8%) were detected by the algorithm. The algorithm suggested a possible missing comorbidity in 2950 of the 4312 stays (68.4%) (Table 1).

The two experts confirmed the suggested diagnoses in 58 of the 285 reviewed stays (Precision, 20.3%) and added 76 ICD-10 codes (Additional file 4). Among these 58 stays, 18 (31.0%) had a major comorbidity added (Table 1) (Additional file 5).

The two experts could not confirm the suggested diagnoses in 227 reviewed stays for two mains reasons: i) the EHR had no information concerning the suggested comorbidity (*n* = 95; 41.9%); ii) the indication for the drug prescription was implicit and therefore could not be added to the stay (*n* = 132; 58.1%).

Combining SPS prescriptions and laboratory results (plasma potassium concentration) improved the detection of hyperkalemia by 66.8%, compared with using only the laboratory results. Specifically, 1270 stays in the general dataset had laboratory results including blood potassium values and in 152 of them (12%) this value was higher than 5 mmol/L, but without a hyperkalemia ICD-10 billing code. This means that for 12% of stays in this dataset the hyperkalemia ICD-10 code could be added only on the basis of the laboratory test results. Moreover, 299 stays in this dataset included an SPS prescription. Among them, 85 did not have a hyperkalemia ICD-10 billing code and 67 had a potassium concentration value higher than 5 mmol/L, which means that in 78.8% (67/85) of stays, the algorithm could detect a hyperkalemia ICD-10 code by combining the laboratory test result and the SPS prescription.

## Discussion

This study demonstrates that an algorithm based on clinical data and knowledge databases can detect comorbidities in EHR. The algorithm suggested missing comorbidities for more than half of the stays and, depending on the dataset, billing codes were improved by 6–8% after expert review. Moreover, by combining drug prescriptions and laboratory test results hyperkalemia detection was improved by 67%.

To develop this algorithm, we applied strategies similar to those used for adverse drug event detection [20–22]. First, we enriched a pharmaceutical database with a comorbidity list. Indeed, 90% of drugs in the Theriaque database have at least one ICD-10 drug indication. However, only 11% of the existing billing codes are present in the Theriaque database. On the other hand, the algorithm cannot use drugs with a wide range of indications (up to hundred codes) because they cannot be associated with a limited list of comorbidities. Moreover, in specialties, such as psychiatry and rheumatology, most drugs are prescribed to treat symptoms rather than specific diseases. In addition, some major comorbidities from the French Comorbidity List are treated surgically (e.g., gastrocolic fistulas [23]) and therefore cannot be detected with this algorithm. Despite these limitations, the algorithm covers 75% of stays with at least one drug prescription and about half of all hospital stays, confirming that drugs are adequate to capture comorbidities.

Then, we combined laboratory test results and prescription of drugs with narrow indications (SPS, in this study) to improve comorbidity detection [24]. This result needs to be confirmed in other similar situations, to further validate the algorithm performance.

We then used data extracted from the EHOP CDW to confirm the diagnoses suggested by the algorithm. The EHOP CDW gives the opportunity to get back to the free text reports (unstructured data) [13]. This allowed us not only to identify relevant information with the help of rules based on structured data, but also to validate and ensure the accuracy of such information. The suggested codes were always manually validated to confirm their accuracy and relevance. Indeed, the objective our system is not to automatically add codes to the EHR, but rather to highlight patient records that could be improved without the need to systematically review all records.

Although the algorithm can identify missing comorbidities, the method has some limitations. First, some drug prescriptions are not covered by the Theriaque database, because it only records indications approved by regulatory agencies for the drug original marketing authorizations, but not “off-label” usage [25]. Yet, doctors do prescribe “off-label” and regulatory agencies recognize this use for patient groups not included in the original approval [26]. As a result, several discrepancies exist between the “official” indication for a drug in the Theriaque database and the actual reasons for its prescription. The algorithm cannot detect these situations because it only relies on official drug indications.

Second, the Cohen’s kappa coefficient value (0.74) indicates a substantial agreement between observers, which could be explained by their common background and similar training. This value could have been improved by involving more human experts, but very few were available at the time of the study.

Furthermore, the suggested diagnoses were confirmed only in 20% of cases. The remaining diagnoses could not be confirmed mainly because information was often implicit, a recurring issue in healthcare [27], particularly in the case of preventive treatments. For example, secondary prevention after a cardiac event is an essential part of cardiovascular disease management and is integrated in the everyday practice, as recommended by clinical guidelines [28]. Thus, for patients with cardiovascular diseases, physicians do not explicitly write down the arguments for their prescription in their notes, because they expect other physicians to understand the initial reasoning by (i) reading the patient’s medical history, and (ii) by consulting the official guideline practices related to that condition. Here, the algorithm suggested a disease, while the clinical notes mentioned a risk factor rather than a comorbid condition. To improve the algorithm limitations concerning implicit information, the use of natural language processing (NLP) methods could be explored. Indeed, NLP methods can identify implicit information in medical notes [29] using machine learning-based [30], rule-based [31] or both approaches [32, 33]. Although NLP methods yield good results [29, 34], they often target specific diseases, and it would be hard to differentiate between main disease and comorbidities. Future studies should determine whether combining our approach with NLP methods and focusing on the most frequently missing comorbidities might improve the algorithm performance [35].

Nevertheless, our algorithm can improve the patients’ characterization using EHR data [36]. This is important because patient phenotyping [37] is now employed to describe patients not only for optimal reimbursement [38], but also for research purposes [36, 39]. EHR-driven phenotyping is efficient only if ICD-10 billing codes are sufficiently exhaustive [40]. Our algorithm adds another level of information to the clinical data by using knowledge databases. It improves the reliability of the ICD-10 billing codes used to describe patients. We think that this could help ensuring accurate reimbursement of patient stays and improve knowledge discovery from studies based on EHR data. The next step in our project is to ask clinical coders to test the algorithm in the daily practice. We think that they could use the algorithm to improve their initial coding from scratch and we would like to fully implement it in the EHOP CDW. In the future, we want to automatically send to the coding software the list of stays with potentially missing comorbidities and the codes suggested by the algorithm. Thus, all clinical coders will benefit from the system for their daily practice.

## Conclusions

We developed a fairly simple algorithm based on accessible and immediately reusable data. This study highlights that knowledge databases, drug prescriptions and laboratory test results can be used together to improve comorbidity detection. Combining this approach with NLP methods focused on implicit information should improve the algorithm performance.

## Additional files

Additional file 1: List of excluded drugs with broad or imprecise indications according to their Anatomical Therapeutic Chemical (ATC) class. This table lists all drugs that were not included in the first step of the algorithm, with their ATC code and label. (DOCX 12 kb)

Additional file 2: Algorithm Evaluation: Inter-observer agreement process. This file explains in detail inter-observer agreement process: the data used, all the different steps, the formalization, the process in case of disagreement between the experts. (DOCX 19 kb)

Additional file 3: Characteristics of all ICD-10 codes suggested by the Theriaque database. This table describes the aggregated characteristics of all ICD-10 codes suggested by the algorithm for each dataset. The codes are separated between “Match” codes (codes added after expert review) and “Non-match” codes (codes that could not been added after expert review). (DOCX 15 kb)

Additional file 4: Characteristics of the ICD-10 codes added after expert review. This table describes the aggregated characteristics of the ICD-10 codes added to each dataset after the manual review by the two experts. It also count the number of ICD-10 codes that were part of the CMA list. (DOCX 13 kb)

Additional file 5: List of the ICD-10 codes added after expert review. This table lists all ICD-10 codes that were added after expert review, the number of stays were they were added, and specify if these codes are part of the CMA list. (DOCX 17 kb)

Additional file 6: Ethics committee approval. This file (in French) is the official ethics committee approval for the study. (DOC 85 kb)

## Abbreviations

ATC: Anatomical therapeutic chemical terminology
CDW: Clinical data warehouse
CMA: French comorbidity list
CUD: Common units of dispensation
DRG: Diagnosis-related groups
EHR: Electronic health records
ENT: Ear; Nose, Throat
ICD-10: International classification of diseases, 10th edition
NLP: Natural language processing
RUH: Rennes university hospital
SPS: Sodium polystyrene sulfonate
κ: Cohen’s Kappa coefficient

## References

[1] Slee DA, Slee VN, Joachim Schmidt H (2007). Slee’s health care terms 5th edition.

[2] O’Leary KJ, Devisetty VK, Patel AR, Malkenson D, Sama P, Thompson WK (2013). Comparison of traditional trigger tool to data warehouse based screening for identifying hospital adverse events. BMJ Qual Saf.

[3] Prokosch HU, Ganslandt T (2009). Perspectives for medical informatics. Reusing the electronic medical record for clinical research. Methods Inf Med.

[4] Ladha KS, Eikermann M (2015). Codifying healthcare--big data and the issue of misclassification. BMC Anesthesiol.

[5] Januel J-M, Luthi J-C, Quan H, Borst F, Taffé P, Ghali WA (2011). Improved accuracy of co-morbidity coding over time after the introduction of ICD-10 administrative data. BMC Health Serv Res.

[6] ATIH: Agence technique de l’information sur l’hospitalisation [Internet]. [cited 2015 Apr 20]. Available from: http://www.atih.sante.fr/

[7] Quan H, Parsons GA, Ghali WA (2002). Validity of information on comorbidity derived rom ICD-9-CCM administrative data. Med Care.

[8] Quan H, Sundararajan V, Halfon P, Fong A, Burnand B, Luthi J-C (2005). Coding algorithms for defining comorbidities in ICD-9-CM and ICD-10 administrative data. Med Care.

[9] Murphy SN, Weber G, Mendis M, Gainer V, Chueh HC, Churchill S (2010). Serving the enterprise and beyond with informatics for integrating biology and the bedside (i2b2). J Am Med Inform Assoc.

[10] Cuggia M, Garcelon N, Campillo-Gimenez B, Bernicot T, Laurent J-F, Garin E (2011). Roogle: an information retrieval engine for clinical data warehouse. Stud Health Technol Inform.

[11] Xie M, Weinger MB, Gregg WM, Johnson KB (2014). Presenting multiple drug alerts in an ambulatory electronic prescribing system: a usability study of novel prototypes. Appl Clin Inform.

[12] Faustini A, Canova C, Cascini S, Baldo V, Bonora K, De Girolamo G (2012). The reliability of hospital and pharmaceutical data to assess prevalent cases of chronic obstructive pulmonary disease. COPD.

[13] Delamarre D, Bouzille G, Dalleau K, Courtel D, Cuggia M (2015). Semantic integration of medication data into the EHOP clinical data warehouse. Stud Health Technol Inform.

[14] Husson M-C (2008). Theriaque: independent-drug database for good use of drugs by health practitioners. Ann Pharm Fr.

[15] Hacin L, Mainar A, Édouard B (2013). Assessment of pharmaceutical databases available in France. Ann Pharm Fr.

[16] WHOCC - Structure and principles [Internet]. [cited 2016 Nov 1]. Available from: http://www.whocc.no/atc/structure_and_principles/

[17] Liu Z, Guo F, Gu J, Wang Y, Li Y, Wang D (2015). Similarity-based prediction for anatomical therapeutic chemical classification of drugs by integrating multiple data sources. Bioinformatics.

[18] Collège Universitaire des Enseignants de Néphrologie (2016). Néphrologie.

[19] R Core Team (2016). R: a language and environment for statistical computing.

[20] King WJ, Paice N, Rangrej J, Forestell GJ, Swartz R (2003). The effect of computerized physician order entry on medication errors and adverse drug events in pediatric inpatients. Pediatrics.

[21] Mekhjian HS, Kumar RR, Kuehn L, Bentley TD, Teater P, Thomas A (2002). Immediate benefits realized following implementation of physician order entry at an academic medical center. J Am Med Inform Assoc.

[22] Bonnabry P, Despont-Gros C, Grauser D, Casez P, Despond M, Pugin D (2008). A risk analysis method to evaluate the impact of a computerized provider order entry system on patient safety. J Am Med Inform Assoc.

[23] Yin J, Zheng Z, Cai J, Song J, Wang J, Zhang J (2014). Current diagnosis and management of malignant gastrocolic fistulas: a single surgical unit’s experience. Int J Clin Exp Med.

[24] Ficheur G, Chazard E, Beuscart J-B, Merlin B, Luyckx M, Beuscart R (2014). Adverse drug events with hyperkalaemia during inpatient stays: evaluation of an automated method for retrospective detection in hospital databases. BMC Med Inform Decis Mak.

[25] Wittich CM, Burkle CM, Lanier WL (2012). Ten common questions (and their answers) about off-label drug use. Mayo Clin Proc.

[26] Elias M, Monique M, Tom W (2004). Regulating pharmaceuticals in Europe: striving for efficiency, equity and quality: striving for efficiency, equity and quality.

[27] Hanlon JT, Schmader KE (2013). The medication appropriateness index at 20: where it started, where it has been, and where it may be going. Drugs Aging.

[28] Smith SC, Benjamin EJ, Bonow RO, Braun LT, Creager MA, Franklin BA (2011). AHA/ACCF secondary prevention and risk reduction therapy for patients with coronary and other atherosclerotic vascular disease: 2011 update: a guideline from the American Heart Association and American College of Cardiology Foundation. Circulation.

[29] Uzuner O, Goldstein I, Luo Y, Kohane I (2008). Identifying patient smoking status from medical discharge records. J Am Med Inform Assoc.

[30] Wicentowski R, Sydes MR (2008). Using implicit information to identify smoking status in smoke-blind medical discharge summaries. J Am Med Inform Assoc.

[31] Wang Y, Chen ES, Leppik I, Pakhomov S, Sarkar IN, Melton GB (2016). Identifying family history and substance use associations for adult epilepsy from the electronic health record. AMIA Jt Summits Transl Sci Proc.

[32] Chen Q, Li H, Tang B, Wang X, Liu X, Liu Z (2015). An automatic system to identify heart disease risk factors in clinical texts over time. J Biomed Inform.

[33] Urbain J (2015). Mining heart disease risk factors in clinical text with named entity recognition and distributional semantic models. J Biomed Inform.

[34] Uzuner O (2009). Recognizing obesity and comorbidities in sparse data. J Am Med Inform Assoc.

[35] Ford E, Nicholson A, Koeling R, Tate A, Carroll J, Axelrod L (2013). Optimising the use of electronic health records to estimate the incidence of rheumatoid arthritis in primary care: what information is hidden in free text?. BMC Med Res Methodol.

[36] Peissig PL, Santos Costa V, Caldwell MD, Rottscheit C, Berg RL, Mendonca EA (2014). Relational machine learning for electronic health record-driven phenotyping. J Biomed Inform.

[37] Wojczynski MK, Tiwari HK (2008). Definition of phenotype. Adv Genet.

[38] Palmer KS, Agoritsas T, Martin D, Scott T, Mulla SM, Miller AP (2014). Activity-based funding of hospitals and its impact on mortality, readmission, discharge destination, severity of illness, and volume of care: a systematic review and meta-analysis. PLoS One.

[39] Kho AN, Pacheco JA, Peissig PL, Rasmussen L, Newton KM, Weston N (2011). Electronic medical records for genetic research: results of the eMERGE consortium. Sci Transl Med.

[40] Newton KM, Peissig PL, Kho AN, Bielinski SJ, Berg RL, Choudhary V (2013). Validation of electronic medical record-based phenotyping algorithms: results and lessons learned from the eMERGE network. J Am Med Inform Assoc.

