# Supplementary material for: Combining information from a clinical data warehouse and a pharmaceutical database to generate a framework to detect comorbidities in electronic health records
Source: BMC Med Inform Decis Mak. 2018 Jan 24;18:9. doi: 10.1186/s12911-018-0586-x (PMC5784648; doi:10.1186/s12911-018-0586-x)
Supplement: Supplementary file 1 — List of excluded drugs with broad or imprecise indications according to their Anatomical Therapeutic Chemical (ATC) class. This table lists all drugs that were not included in the first step of the algorithm, with their ATC code and label. (DOCX 12 kb) [file 12911_2018_586_MOESM1_ESM.docx]

**Additional file 1: List of excluded drugs with broad or imprecise indications according to their Anatomical Therapeutic Chemical (ATC) class**

| **ATC Class Code** | **ATC Class Label** |
| --- | --- |
| A03A | Drugs for functional gastrointestinal disorders |
| A03BA | Belladonna alkaloids, tertiary amines |
| A03C | Antispasmodics in combination with psycholeptics |
| A03D | Antispasmodics in combination with analgesics |
| A03F | Propulsives |
| A04 | Antiemetics and antinauseants |
| A06A | Drugs for constipation |
| B05B | Intravenous solutions |
| C10 | Lipid modifying agents |
| J01 | Antibacterials for systemic use |
| J07 | Vaccines |
| M01AB | Acetic acid derivatives and related substances |
| M01AE | Propionic acid derivatives |
| M01AG | Fenamates |
| M01AH | COX-2 inhibitors |
| M02 | Topical products for joint and muscular pain |
| N01 | Anesthetics |
| N02 | Analgesics |
| N05B | Anxiolytics |
| N05C | Hypnotics and sedatives |
| R05D | Cough suppressants, excluding combinations with expectorants |
| R05F | Cough suppressants and expectorants, combinations |
| R05X | Other cold preparations |
