# Supplementary material for: Combining information from a clinical data warehouse and a pharmaceutical database to generate a framework to detect comorbidities in electronic health records
Source: BMC Med Inform Decis Mak. 2018 Jan 24;18:9. doi: 10.1186/s12911-018-0586-x (PMC5784648; doi:10.1186/s12911-018-0586-x)
Supplement: Supplementary file 2 — Algorithm Evaluation: Inter-observer agreement process. This file explains in detail inter-observer agreement process: the data used, all the different steps, the formalization, the process in case of disagreement between the experts. (DOCX 19 kb) [file 12911_2018_586_MOESM2_ESM.docx]

**Additional file 2: Algorithm Evaluation: Inter-observer agreement process**

The reference file is a list of stays where the prescribed drugs were matched with the drug indications (ICD-10 code), according to the Theriaque database. If these ICD-10 codes were included in the Comorbidity List, they were flagged as possible comorbidities. Each expert also had full access to the last digital version of the French ICD-10 provided by The Program of Medicalization of Information Systems.

- Confirmation of the suggested diagnoses by the medical expert using information from the EHOP Clinical Data Warhouse (CDW)

A de-identified datamart was created with only the stays that needed to be evaluated. Each medical expert had independent access to the datamart and had to log-in in the hospital system to access the data.

For each stay:

1. The expert needed to confirm at least one of the suggested comorbidities. He used the full text search function available in the CDW:
   1. First, by looking for the full label of the ICD-10 code (e.g., «Non-traumatic extradural hemorrhage»)
   2. If no result was retrieved, the experts looked for the short version of the label («Extradural hemorrhage»)
   3. If the expert could not find anything, he looked for the synonyms provided by ICD-10 (« Non-traumatic epidural hemorrhage»)
   4. If the expert still could not find anything in the EHR, the suggested diagnosis was flagged as «**not** found» and the expert could move to the next suggested diagnosis.
2. If the expert could confirm a suggested code based on the EHR, he did not have to look for other suggestions because the consensus was that one drug was prescribed for one disease.
3. If the expert could not confirm a suggested code, he had to read the entire discharge note to confirm that this was due to the absence of information on that disease, rather than to a weakness of the CDW search tool.
4. Once all prescriptions for a stay were reviewed, the expert could move to the next stay.

Codes were reviewed by order of severity, according to the Comorbidity List (from most to least severe comorbidity).

- Results and data formatting
- The column «Confirmed Diagnosis» (**Format: yes/no**): it specifies whether one of the suggested diagnoses was confirmed by the EHR review.
- The suggested diagnosis that could be confirmed was flagged when the column “Confirmed diagnosis” was filled with a “yes”.
- If the column «Confirmed Diagnosis» **(Format: 1/2/3/4**) was filled with a no, the expert had to fill in the column «Non-confirmed diagnosis» to clarify why he/she could not confirm at least one of the suggested diagnoses. There were four case scenarios and four options:

1. The expert could not find anything in the EHR to confirm this diagnosis despite his/her thorough research.
2. The suggested code was a symptom of a disease/syndrome already coded by the clinical coder. This can happen because of the French coding guidelines (if the patient has several symptoms that are all part of a unique disease/syndrome, the coder must only code the disease and cannot code the symptoms).
3. The expert could find a diagnosis, but it was not the exact diagnosis suggested by the system («Imprecise diagnosis»)
4. The diagnosis could not be confirmed for “Other” reasons.

- The «Imprecise Diagnosis» column (**Format: ICD-10 code**): The expert had to fill in this column if he chose the option «3» in the «Non-confirmed Diagnosis».

In this case, the system suggested a code, but due to lack of details in the EHR, the expert could not add this exact code, but only a more general one (same disease, but with the mention «unspecified»).

- The «Comments» column (**Format: free text**): The expert had to fill in this column when choosing option «4» in the «Non-confirmed Diagnosis».

This column allowed the expert to explain why he chose the «Other» category.

A few examples of «Other» explanations:

**Preventive**: This is a preventive treatment, not an actual disease (very common in cardiovascular disease management)

**Implicit**: The reason for the drug prescription is not explicitly written down in the clinical note. Alternatively, all symptoms related to the actual disease are described, but the connection between symptoms and disease is not explicitly written down (although a health professional can make this connection by reading the notes). The coding guidelines do not allow coding a disease if it is not explicitly written down, although all its symptoms are described in the clinical notes.
