# Supplementary material for: Combining information from a clinical data warehouse and a pharmaceutical database to generate a framework to detect comorbidities in electronic health records
Source: BMC Med Inform Decis Mak. 2018 Jan 24;18:9. doi: 10.1186/s12911-018-0586-x (PMC5784648; doi:10.1186/s12911-018-0586-x)
Supplement: Supplementary file 3 — Characteristics of all ICD-10 codes suggested by the Theriaque database. This table describes the aggregated characteristics of all ICD-10 codes suggested by the algorithm for each dataset. The codes are separated between “Match” codes (codes added after expert review) and “Non-match” codes (codes that could not been added after expert review). (DOCX 15 kb) [file 12911_2018_586_MOESM3_ESM.docx]

**Additional file 3: Characteristics of all ICD-10 codes suggested by the Theriaque database**

| **ICD-10 Chapter** | **ENT dataset [N(%)]** | | | **General dataset [N(%)]** | |
| --- | --- | --- | --- | --- | --- |
|  | **Match***  **(n=58)** | **Non match****  **(n=952)** | | **Match***  **(n=76)** | **Non match****  **(n=4551)** |
| Chapter I: Certain infectious and parasitic diseases | - | | 63 (6.6) | 1 (2.6) | 176 (3.9) |
| Chapter III: Diseases of the blood and blood-forming organs and certain disorders involving the immune mechanism | 1 (1.7) | | 10 (1) |  | 31 (0.7) |
| Chapter IV: Endocrine, nutritional and metabolic diseases | 8 (13.8) | | 174 (18.3) | 7 (18.4) | 128 (2.8) |
| Chapter V: Mental and behavioral disorders | 2 (3.4) | | 128 (13.4) | 5 (13.2) | 1106 (24.3) |
| Chapter VI: Diseases of the nervous system | - | | 29 (3) | 1 (2.6) | 7 (0.1) |
| Chapter VII: Diseases of the eye and adnexa | 2 (3.4) | | 10 (1) | - | 8 (0.2) |
| Chapter VIII: Diseases of the ear and mastoid process | - | | 25 (2.6) | - | - |
| Chapter IX: Diseases of the circulatory system | 24 (41.4) | | 99 (10.4) | 9 (23.7) | 795 (17.5) |
| Chapter X: Diseases of the respiratory system | 3 (5.2) | | 35 (3.7) | - | 20 (0.4) |
| Chapter XI: Diseases of the digestive system | 1 (1.7) | | 73 (7.7) | 4 (10.5) | 947 (20.8) |
| Chapter XII: Diseases of the skin and subcutaneous tissue | - | | 33 (3.5) | 1 (2.6) | 34 (0.7) |
| Chapter XIII: Diseases of the musculoskeletal system and connective tissue | 3 (5.2) | | 97 (10.2) | 3 (7.9) | 1144 (25.1) |
| Chapter XIV: Diseases of the genitourinary system | 2 (3.4) | | 23 (2.4) | 1 (2.6) | 41 (0.9) |
| Chapter XV: Pregnancy, childbirth and the puerperium | - | | 5 (0.5) | 1 (2.6) | - |
| Chapter XVI: Certain conditions originating in the perinatal period | - | | 29 (3) | - | 2 (0) |
| Chapter XVII: Congenital malformations, deformations and chromosomal abnormalities | 1 (1.7) | | 1 (0.1) | 1 (2.6) | 80 (1.8) |
| Chapter XVIII: Symptoms, signs and abnormal clinical and laboratory findings, not elsewhere classified | 1 (1.7) | | 97 (10.2) | 1 (2.6) | 16 (0.3) |
| Chapter XIX: Injury, poisoning and certain other consequences of external causes | 2 (3.4) | | 21 (2.2) | - | 16 (0.3) |
| Chapter XXI: Factors influencing health status and contact with health services | 8 (13.8) | | - | 3 (7.9) | - |

ENT: Ear, Nose, Throat; ICD-10: International Classification of Diseases, 10^th^ revision; CMA: French Comorbidity List

*Match: The suggested code was added to the Electronic Health Record after expert review

**Non-match: The suggested code could not be added to the Electronic Health Record after expert review
