# Supplementary material for: Combining information from a clinical data warehouse and a pharmaceutical database to generate a framework to detect comorbidities in electronic health records
Source: BMC Med Inform Decis Mak. 2018 Jan 24;18:9. doi: 10.1186/s12911-018-0586-x (PMC5784648; doi:10.1186/s12911-018-0586-x)
Supplement: Supplementary file 4 — Characteristics of the ICD-10 codes added after expert review. This table describes the aggregated characteristics of the ICD-10 codes added to each dataset after the manual review by the two experts. It also count the number of ICD-10 codes that were part of the CMA list. (DOCX 13 kb) [file 12911_2018_586_MOESM4_ESM.docx]

**Additional file 4: Characteristics of the ICD-10 codes added after expert review**

| **ICD-10 Chapter** | **ENT dataset [N(%)]** | | **General dataset [N(%)]** | |
| --- | --- | --- | --- | --- |
|  | **ICD-10 code**  **(n=58)** | **CMA code**  **(n=12)** | **ICD-10 code**  **(n=76)** | **CMA code**  **(n=29)** |
| Chapter I: Certain infectious and parasitic diseases |  |  | 1 (2.6) | 1 (7.1) |
| Chapter III: Diseases of the blood and blood-forming organs and certain disorders involving the immune mechanism | 1 (1.7) | - | - | - |
| Chapter IV: Endocrine, nutritional and metabolic diseases | 8 (13.8) | 2 (16.7) | 7 (18.4) | 3 (21.4) |
| Chapter V: Mental and behavioral disorders | 2 (3.4) | 1 (8.3) | 5 (13.2) | 1 (7.1) |
| Chapter VI: Diseases of the nervous system | - | - | 1 (2.6) | 1 (7.1) |
| Chapter VII: Diseases of the eye and adnexa | 2 (3.4) | - | - | - |
| Chapter IX: Diseases of the circulatory system | 24 (41.4) | 6 (50) | 9 (23.7) | 5 (35.7) |
| Chapter X: Diseases of the respiratory system | 3 (5.2) | - | - | - |
| Chapter XI: Diseases of the digestive system | 1 (1.7) | - | 4 (10.5) | 2 (14.3) |
| Chapter XII: Diseases of the skin and subcutaneous tissue | - | - | 1 (2.6) | - |
| Chapter XIII: Diseases of the musculoskeletal system and connective tissue | 3 (5.2) | 2 (16.7) | 3 (7.9) | 1 (7.1) |
| Chapter XIV: Diseases of the genitourinary system | 2 (3.4) | - | 1 (2.6) | - |
| Chapter XV: Pregnancy, childbirth and the puerperium | - | - | 1 (2.6) | - |
| Chapter XVII: Congenital malformations, deformations and chromosomal abnormalities | 1 (1.7) | - | 1 (2.6) | - |
| Chapter XVIII: Symptoms, signs and abnormal clinical and laboratory findings, not elsewhere classified | 1 (1.7) | - | 1 (2.6) | - |
| Chapter XIX: Injury, poisoning and certain other consequences of external causes | 2 (3.4) | - | - | - |
| Chapter XXI: Factors influencing health status and contact with health services | 8 (13.8) | 1 (8.3) | 3 (7.9) | - |

ENT: Ear, Nose, Throat; ICD-10: International Classification of Diseases, 10^th^ revision; CMA: French Comorbidity List
