# Supplementary material for: Combining information from a clinical data warehouse and a pharmaceutical database to generate a framework to detect comorbidities in electronic health records
Source: BMC Med Inform Decis Mak. 2018 Jan 24;18:9. doi: 10.1186/s12911-018-0586-x (PMC5784648; doi:10.1186/s12911-018-0586-x)
Supplement: Supplementary file 5 — List of the ICD-10 codes added after expert review. This table lists all ICD-10 codes that were added after expert review, the number of stays were they were added, and specify if these codes are part of the CMA list. (DOCX 17 kb) [file 12911_2018_586_MOESM5_ESM.docx]

**Additional file 5: List of the ICD-10 codes added after expert review**

| **ICD-10**  **Code** | | **ICD-10 Title** | **ENT dataset** | | **General dataset** | |
| --- | --- | --- | --- | --- | --- | --- |
|  |  |  | **Stays*** | **CMA** | **Stays** | **CMA** |
| B95.6 | | Staphylococcus aureus as the cause of diseases classified to other chapters | - | - | 1 | yes |
| D57.1 | | Sickle-cell anemia without crisis | 1 | no | - | - |
| E03.8 | | Other specified hypothyroidism | - | - | 2 | no |
| E03.9 | | Hypothyroidism, unspecified | 1 | no | 1 | no |
| E05.9 | | Thyrotoxicosis, unspecified | 1 | yes | - | - |
| E11.80 | | Non-insulin-dependent diabetes mellitus: With unspecified complications | 1 | yes | - | - |
| E11.90 | | Non-insulin-dependent diabetes mellitus: Without complications | - | - | 1 | yes |
| E13.9 | | Other specified diabetes mellitus: Without complications | 1 | no | - | - |
| E78.5 | | Hyperlipidemia, unspecified | 1 | no | - | - |
| E87.08 | | Hyperosmolality and hypernatremia | - | - | 1 | yes |
| E87.58 | | Hyperkalemia | - | - | 8 | yes |
| E87.68 | | Hypokalemia | 1 | no | 3 | no |
| E89.0 | | Postprocedural hypothyroidism | 2 | no | 1 | no |
| F03 | | Unspecified dementia | 1 | yes | - | - |
| F10.4 | | Mental and behavioral disorders due to use of alcohol: Withdrawal state with delirium | - | - | 1 | yes |
| F31.9 | | Bipolar affective disorder, unspecified | - | - | 2 | no |
| F32.9 | | Depressive episode, unspecified | 1 | no | 1 | no |
| F33.9 | | Recurrent depressive disorder, unspecified | - | - | 7 | no |
| F41.9 | | Anxiety disorder, unspecified | - | - | 2 | no |
| G20 | | Parkinson disease | - | - | 1 | yes |
| H40.2 | | Primary angle-closure glaucoma | 1 | no | - | - |
| H40.8 | | Other glaucoma | 1 | no | - | - |
| I10 | | Essential (primary) hypertension | 11 | no | 3 | no |
| I11.0 | | Hypertensive heart disease with (congestive) heart failure | 2 | yes | - | - |
| I24.8 | Other forms of acute ischemic heart disease | | 1 | no | - | - |
| I25.2 | Old myocardial infarction | | 3 | no | 6 | no |
| I25.9 | Chronic ischemic heart disease, unspecified | | - | - | 2 | no |
| I27.2 | Other secondary pulmonary hypertension | | 1 | yes | - | - |
| I48.9 | Atrial fibrillation and flutter | | 2 | yes | 2 | yes |
| I50.0 | Congestive heart failure | | 1 | yes | 3 | yes |
| I50.1 | Left ventricular failure | | - | - | 1 | yes |
| I50.9 | Heart failure, unspecified | | - | - | 3 | yes |
| I70.20 | Atherosclerosis of arteries of extremities | | - | - | 1 | no |
| I73.0 | Raynaud syndrome | | 1 | no | - | - |
| I73.9 | Peripheral vascular disease, unspecified | | 2 | no | - | - |
| I95.1 | Orthostatic hypotension | | - | - | 1 | yes |
| J34.8 | Other specified disorders of nose and nasal sinuses | | 1 | no | - | - |
| J45.9 | Asthma, unspecified | | 2 | no | - | - |
| K21.9 | Gastro-esophageal reflux disease without esophagitis | | - | - | 5 | no |
| K22.1 | Ulcer of esophagus | | - | - | 1 | yes |
| K44.9 | Diaphragmatic hernia without obstruction or gangrene | | 1 | no | - | - |
| K51.8 | Other ulcerative colitis | | - | - | 1 | no |
| K71.0 | Toxic liver disease with cholestasis | | - | - | 1 | yes |
| L40.0 | Psoriasis vulgaris | | - | - | 1 | no |
| M06.8 | Other specified rheumatoid arthritis | | - | - | 1 | no |
| M10.9 | Gout, unspecified | | 2 | yes | 4 | yes |
| M45+9 | Ankylosing spondylitis | | - | - | 1 | no |
| M81.0 | Postmenopausal osteoporosis | | 1 | no | - | - |
| N18.3 | Chronic kidney disease, stage 3 | | - | - | 1 | no |
| N40 | Hyperplasia of prostate | | 2 | no | - | - |
| O13 | Gestational [pregnancy-induced] hypertension without significant proteinuria | | - | - | 1 | no |
| Q27.3 | Peripheral arteriovenous malformation | | 1 | no | - | - |
| Q40.1 | Congenital hiatus hernia | | - | - | 1 | no |
| R12 | Heartburn | | - | - | 1 | no |
| R79.0 | Abnormal level of blood mineral | | 1 | no | - | - |
| T90.9 | Sequelae of unspecified injury of head | | 2 | no | - | - |
| Z85.5 | Personal history of malignant neoplasm of urinary tract | | 2 | no | - | - |
| Z85.800 | Personal history of malignant neoplasms of other organs and systems | | 1 | no | - | - |
| Z86.70 | Personal history of diseases of the circulatory system | | 2 | no | 1 | no |
| Z95.2 | Presence of prosthetic heart valve | | 1 | yes | - | - |
| Z95.5 | Presence of coronary angioplasty implant and graft | | - | - | 1 | no |
| Z95.80 | Presence of other cardiac and vascular implants and grafts | | - | - | 1 | no |
| Z95.880 | Presence of other cardiac and vascular implants and grafts | | 1 | no | - | - |
| Z96.6 | Presence of orthopedic joint implants | | 1 | no | - | - |

***Stays**: Number of stays where the ICD-10 code was found. ENT: Ear, Nose, Throat; ICD-10: International Classification of Diseases, 10^th^ revision. CMA: French Comorbidity List.
