# Supplementary material for: Combining information from a clinical data warehouse and a pharmaceutical database to generate a framework to detect comorbidities in electronic health records
Source: BMC Med Inform Decis Mak. 2018 Jan 24;18:9. doi: 10.1186/s12911-018-0586-x (PMC5784648; doi:10.1186/s12911-018-0586-x)
Supplement: Supplementary file 6 — Ethics committee approval. This file (in French) is the official ethics committee approval for the study. (DOC 85 kb) [file 12911_2018_586_MOESM6_ESM.doc]

| 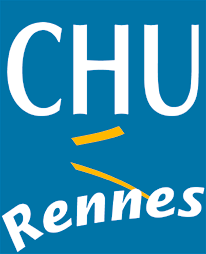 |
| --- |
| **comite d’ethique du CHU** |

Dr Vincent MOREL

Praticien Hospitalier

🕾 02 99 87 35 53

Fax 02 99 87 35 54

[comite.ethique@chu-rennes.fr](mailto:comite.ethique@chu-rennes.fr)

**Dr SYLVESTRE Emmanuelle**

**Interne de santé publique en informatique médicale**

**Pontchaillou**

Avis n° 16.127

Rennes, 04/11/16

Madame et chère Consœur,

Après étude du projet de recherche

**Exploitation d’une base de connaissance médicamenteuse et d’un entrepôt de données pour la détection de comorbidités dans le Dossier Patient Informatisé.**

Le comité d’éthique du CHU de Rennes estime que ce projet de recherche ne contrevient pas à l’éthique médicale.

Cet avis du comité d’éthique a été émis à l’unanimité de ses membres présents.

En vous souhaitant bonne réception.

Veuillez agréer, Madame et chère Consœur, l’expression de ma considération la meilleure.

Pour le comité d’éthique

Vincent Morel
